# Supine Frequent Ventricular Extrasystoles in a Pregnant Woman without Structural Heart Disease

**DOI:** 10.1155/2016/6213198

**Published:** 2016-08-25

**Authors:** Natália Stela Sandes Ferreira, Tatiana La Croix Barros, Ronaldo Altenburg Gismondi

**Affiliations:** Clinical Medicine Department, Antônio Pedro University Hospital, Fluminense Federal University, RJ, Brazil

## Abstract

Arrhythmias are the most common cardiac complication during gestational period and may occur in women with or without known structural heart disease. Premature extra beats and sustained tachyarrhythmias are the most common arrhythmias in pregnancy. Symptomatic episodes occur in 20–44% of pregnant women, usually as palpitations, dizziness, or syncope. We searched on Pubmed for ventricular premature complexes (VPC) in pregnant women and found no case reporting increased incidence of this arrhythmia while supine. The aim of this study is to report a case of a pregnant woman without previous structural heart disease that presented a great number of VPC when supine. The arrhythmogenesis increase during pregnancy is multifactorial. In the reported case, we believe that augmented venous return was the most important pathophysiologic process. When the patient changes to left lateral decubitus, there could be a sudden release of the inferior vena cava, causing an abrupt augmentation of venous return to the right heart chambers and increasing the risk of arrhythmias. Obstetricians and primary care physicians should be aware of palpitations and related patient complains while they are asleep or supine.

## 1. Introduction

Arrhythmias are the most common cardiac complication during gestational period, occurring in up to 50% of pregnant women [1, 2]. Arrhythmia may occur in women with or without known structural heart disease. Cardiovascular adaptations to pregnancy may trigger arrhythmias and/or exacerbate preexisting ones [3]. In the gestational period, total body water increases by 40–50%, therefore expanding blood volume and cardiac output and implying significant mechanical overload on the maternal heart [2, 3]. The cardiac size increases and augments wall tension, stimulating the stretch-activated ion channels in myocytes. Moreover, rest heart rate elevates about 10 to 20 beats, shortening PR, QRS, and QT intervals [4]. Changes in autonomic tone, systemic hemodynamic vasodilation, and mild hypokalemia may contribute to arrhythmogenesis [5].

Premature extra beats and sustained tachyarrhythmias are the most common arrhythmias in pregnancy. Symptomatic episodes occur in 20–44% of pregnant women, usually as palpitations, dizziness, or syncope [1]. Atrial premature complexes (APC), ventricular premature complexes (VPC), and supraventricular tachyarrhythmias are more prevalent than ventricular tachycardia, especially in women without structural heart disease. In a study of 162 pregnant women, symptoms were more frequent when there were more than 50 VPC per 24 hours [1]. However, APC and VPC may also occur without symptoms.

We searched on Pubmed for VPC in pregnant women and found no case reporting increased incidence of this arrhythmia while supine. The aim of this study is to report a case of a pregnant woman without previous structural heart disease that presented a great number of VPC when supine.

## 2. Case Presentation

A 27-year-old white woman reported palpitations and dizziness when supine. She was pregnant and her gestational age was 32 weeks. The patient was previously healthy and denies use of drugs, alcohol, or medicines. There was no history of known structural heart disease, arrhythmias or prior unexplained syncope. The patient was sedentary but did not report any symptoms while on exertion. She did not smoke and coffee ingestion was 100 to 200 mL per day on average. Clinical examination was remarkably normal. At rest, blood pressure was 128/74 mmHg and heart rate was 90 bpm.

**Figure 1:**
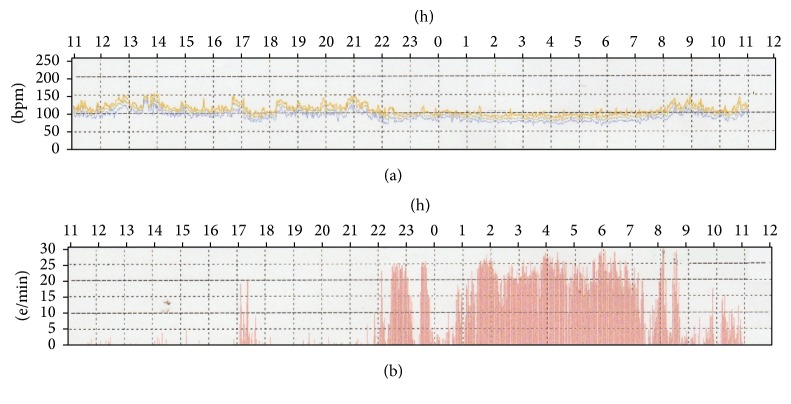
Heart rate (a) and ventricular premature complexes (b) during 24-hour Holter monitoring.

The patient performed laboratory tests and transthoracic echocardiogram; all exams were within normal range (Table 1). A 24-hour Holter monitoring showed high incidence of VPC when the patient lied down to sleep (Figure 1). Frequency of VPC was very low during daytime activities. Treatment with beta-blocker was proposed but patient denied it. We recommended left lateral decubitus while sleeping, but symptoms persisted. Delivery took placed without any complications. Symptoms improved in the puerperal period and the patient and her baby are nowadays doing remarkably well.

## 3. Discussion

We reported a pregnant woman without structural heart disease that had multiple symptomatic VPC when supine. Despite physiological adaptations of the cardiovascular system during gestational period, the hemodynamic, metabolic, and autonomic changes that occur may predispose pregnant women to an increased risk of arrhythmias [2, 3].

VPC are very common in pregnancy, occurring in up to 50% of pregnant women [2]. Although APC and VPC are not associated with increased maternal mortality, symptomatic patients may warrant treatment with beta-blockers [4]. Furthermore, it is important to identify and stop potential precipitating factors, such as alcohol and stimulants use. Previous studies question if reducing caffeine intake diminishes the number of premature cardiac beats [6, 7]. In addition, any woman with arrhythmia while pregnant should be submitted to clinical evaluation looking for structural heart disease. In most cases, electrocardiogram, transthoracic echocardiogram, and 24-hour Holter monitoring are recommended.

The arrhythmogenesis increase during pregnancy is multifactorial [2, 3]. Estrogen upregulation of the myocardial alpha receptors increases adrenergic activity and may enhance both automaticity and triggered activity. In addition, intravascular volume increases, augmenting ventricular preload and therefore ventricular and atrial sizes [8]. Atrial and ventricular stretch may contribute to arrhythmogenesis due to stretch-activated ion channel activity causing membrane despolarization, shortened refractoriness, slowed conduction, and spatial dispersion of refractoriness and conduction [9]. The increase in resting heart rate has also been associated to markers of arrhythmogenesis such as late potentials and depressed heart rate variability [10].

In the reported case, we believe that augmented venous return was the most important pathophysiologic process, but elevated daytime heart rate may also have played a role. Cardiac preload increases when patients lie down due to gravity forces. In pregnancy, peripheral edema is more severe and therefore venous return is increased. This stretches right cardiac chambers and promotes arrhythmias. Another hypothesis is the “inferior cava squeeze”: in supine or in right lateral decubitus, the gravid uterus squeezes the inferior vena cava. When the patient changes to left lateral decubitus, there could be a sudden release of the inferior vena cava, causing an abrupt augmentation of venous return to the right heart chambers and increasing the risk of arrhythmias. The reported patient could not remember her decubitus while asleep, but her obstetrician recommended left lateral decubitus.

A previous study enrolled 110 pregnant symptomatic women without structural heart disease referred for evaluation of syncope, dizziness, or palpitations and compared them to 52 asymptomatic pregnant women referred for cardiac murmurs evaluation [1]. Both groups had high incidence of arrhythmias in 24-hour Holter monitoring (59% and 50%, resp.). The number of VPC was higher in symptomatic patients, but there was poor correlation between VPC frequency and symptoms: only 10% of symptomatic episodes were accompanied by the presence of arrhythmias. Holter monitoring was repeated 6 weeks after delivery in nine women and showed a significant reduction in VPC frequency [1]. In our case, there was a correlation between symptoms and VPC occurrence. We chose to not repeat Holter after delivery because the patient was totally asymptomatic.

In conclusion, pregnant women have higher risk of arrhythmias due to physiological changes in pregnancy. VPC are one of the most common arrhythmia and may occur asymptomatic as well as in patients without structural heart disease. Supine position and changes from left lateral decubitus to right lateral decubitus may precipitate arrhythmias. Obstetricians and primary care physicians should be aware of palpitations and related patient complains while they are asleep or supine.

## Figures and Tables

**Table 1:** Laboratorial parameters.

Parameter	Value
Hemoglobin (g/dL)	12.9
Glucose (mg/dL)	75
Creatinine (mg/dL)	0.78
Sodium (mEq/L)	140
Potassium (mEq/L)	4.1
Uric acid (mg/dL)	2.6
Total cholesterol (mg/dL)	261
LDL-cholesterol (mg/dL)	141
HDL-cholesterol (mg/dL)	80
Triglycerides (mg/dL)	199
TSH (mcUI/mL)	1.12

HDL, high density lipoprotein; LDL, low density lipoprotein; TSH, thyroid stimulating hormone.

## References

[1] Shotan A., Ostrzega E., Mehra A., Johnson J. V., Elkayam U. (1997). Incidence of arrhythmias in normal pregnancy and relation to palpitations, dizziness, and syncope. *The American Journal of Cardiology*.

[2] Adamson D. L., Nelson-Piercy C. (2007). Managing palpitations and arrhythmias during pregnancy. *Heart*.

[3] Burkart T. A., Conti J. B. (2010). Cardiac arrhythmias during pregnancy. *Current Treatment Options in Cardiovascular Medicine*.

[4] Knotts R. J., Garan H. (2014). Cardiac arrhythmias in pregnancy. *Seminars in Perinatology*.

[5] Gowda R. M., Khan I. A., Mehta N. J., Vasavada B. C., Sacchi T. J. (2003). Cardiac arrhythmias in pregnancy: clinical and therapeutic considerations. *International Journal of Cardiology*.

[6] Newby D. E., Neilson J. M. M., Jarvie D. R., Boon N. A. (1996). Caffeine restriction has no role in the management of patients with symptomatic idiopathic ventricular premature beats. *Heart*.

[7] Myers M. G. (1991). Caffeine and cardiac arrhythmias. *Annals of Internal Medicine*.

[8] Franz M. R., Cima R., Wang D., Profitt D., Kurz R. (1992). Electrophysiological effects of myocardial stretch and mechanical determinants of stretch-activated arrhythmias. *Circulation*.

[9] Kamkin A., Kiseleva I., Isenberg G. (2000). Stretch-activated currents in ventricular myocytes: amplitude and arrhythmogenic effects increase with hypertrophy. *Cardiovascular Research*.

[10] Soliman E. Z., Elsalam M. A., Li Y. (2010). The relationship between high resting heart rate and ventricular arrhythmogenesis in patients referred to ambulatory 24 h electrocardiographic recording. *Europace*.

